# Lightweight and Compression-Resistant Carbon-Based Sandwich Honeycomb Absorber with Excellent Electromagnetic Wave Absorption

**DOI:** 10.3390/nano12152622

**Published:** 2022-07-29

**Authors:** Song Bi, Yongzhi Song, Genliang Hou, Hao Li, Nengjun Yang, Zhaohui Liu

**Affiliations:** 1 304 Department, Xi’an Research Institute of High-Tech, Xi’an 710025, China; syz237530783@163.com (Y.S.); hougenliang@163.com (G.H.); 376467729lihao@163.com (H.L.); yangbcpl@163.com (N.Y.); 2 College of Weapon Science and Technology, Xi’an Technological University, Xi’an 710025, China

**Keywords:** HC, CNTs/CB/RGO/PU, microwave-absorption properties, compression stress

## Abstract

Honeycomb (HC) composites were fabricated by impregnating an aramid paper HC core with carbon nanotubes/carbon black/reduced graphene oxide (CNTs/CB/RGO) and polyurethane resin (PU). The sandwich HC (SHC) absorber containing HC composites with superior microwave-absorption properties were fabricated using the vacuum bagging method. Through the absorption performance of the SHC absorber, it can be concluded that the triple-layer SHC absorber has the best absorbing performance. The effective bandwidth (reflection loss < 10 dB) can be achieved in the entire frequency range of 2.2–18 GHz, and the minimum RL value is −35 dB. Furthermore, the compressive stress of the triple-layer SHC absorber reached 3.71 MPa, which is similar to the compressive stress of aluminum HC panels for aviation. Benefiting from the excellent integration of absorption and mechanical performance, the SHC has significant potential in the stealth-technology field.

## 1. Introduction

In the last few decades, carbon-based nanoabsorbers have been extensively studied as fillers for structural absorbers [1,2,3]. Especially for the aerospace industry, where the property of light weight is essential, carbon-based structural absorbers have received much attention because of their thermal stability, light weight, and broadband electromagnetic (EM) wave absorption [4,5,6].

Carbon black (CB), carbon nanotubes (CNTs), and reduced graphene oxide (RGO) are all carbon-based microwave-absorbing materials. However, due to their structural characteristics, they have unique EM wave response behavior. Combining and synergizing their respective properties organically yields dramatic wave-absorption performance. Sun et al. [7] prepared multiscale nitrogen-doped RGO@CNT composites, which facilitated the conversion of EM energy to thermal energy by modulating the material ratio to establish conductive channels. An effective absorption bandwidth of 7.1 GHz was achieved with a maximum reflection loss (RL) of 49.4 dB at an ultralow material loading of 2 wt%. Tang et al. [8] fabricated carbon black–reduced graphene oxide (CB/RGO) composites by a simple synthesis method. The composites exhibited excellent microwave-absorption performance, with a maximum absorption intensity of 47.5 dB at a low filler load of 7 wt%. The effective absorption bandwidth reached 5.92 GHz, covering 37% in 2–18 GHz, when the thickness was only 2.2 mm.

In carbon-based structural absorbers, carbon-based absorbents are generally filled in the frame or attached to the frame surface to play the role of EM wave absorption, such as HC [9], unwoven fabric [10], and foam [11] structural absorbers. The design of the microstructure of the filler material and the absorber’s macrostructure can integrate the inherent material loss and structural resonance effect and effectively enhance the dissipation of EM wave energy [12]. Moreover, to realize the lightweight broadband microwave-absorption structural absorbers, it is necessary to use a structure with a low weight ratio, such as the SHC structure [13,14,15]. Kou et al. [16] used an HC structure filled with porous graphene as a radar absorbing material, and the experiment proved that it had good radar absorption performance. At 8.8 GHz, the RL_min_ of this graphene-based composite was −15 dB, thickness only 4.0 mm, and area density 0.26 kg/m^2^. Wang [17] produced a new paper-based composite (PBC) HC material comprised of chopped carbon fiber, aramid fiber, and aramid raw fiber. The RL of composites with a height of 30 mm was lower than −10 dB in the frequency bands of 2–2.5 GHz and 4.4–18 GHz. Moreover, Birman v [18] discussed the novel design of SHC. By introducing CNTs, almost all mechanical and thermodynamic responses of SHC were enhanced. Therefore, carbon-based SHC absorbers have promising application prospects and high research value.

In this paper, four ratios of acidified CNTs and CB/RGO were dispersed into polyurethane resin (PU) to form a homogeneous slurry. The HC cores were impregnated in the slurry to form four single-layer HC composites. According to the principle of gradient concentration, the single-layer HC composites were bonded to prepare multi-layer HC composites. Analyzing the absorbing properties of HC composites, the HC composites with excellent absorbing properties were selected and prepared into SHC absorbers. The testing results of reflection loss and compression stress show that the SHC absorbers have broadband absorption and strong compression resistance performance.

## 2. Materials and Methods

### 2.1. Materials

Carbon nanotubes (CNTs, multiwalled, diameter 1–2 nm, length 5–30 μm), carbon black (CB) were purchased from Suzhou First Element Nanotechnology Co., Ltd., Jiangsu, China. Aramid HC core (Nom-ex, 180 mm × 180 mm × 5 mm, side length 2.75 mm), waterborne polyurethane resin (PU, NL-236, 30%), fiberglass board (180 mm × 180 mm × 0.5 mm), carbon fiber board (180 mm × 180 mm × 0.5 mm), prepreg, film, waterborne wetting dispersant (P-712), and waterborne antifoaming agent (SP-852) were provided by Wuhan Magnetism-Electron Company, Hubei, China.

### 2.2. Preparation of CNT/CB/RGO/PU HC Composites

**Figure 1:**
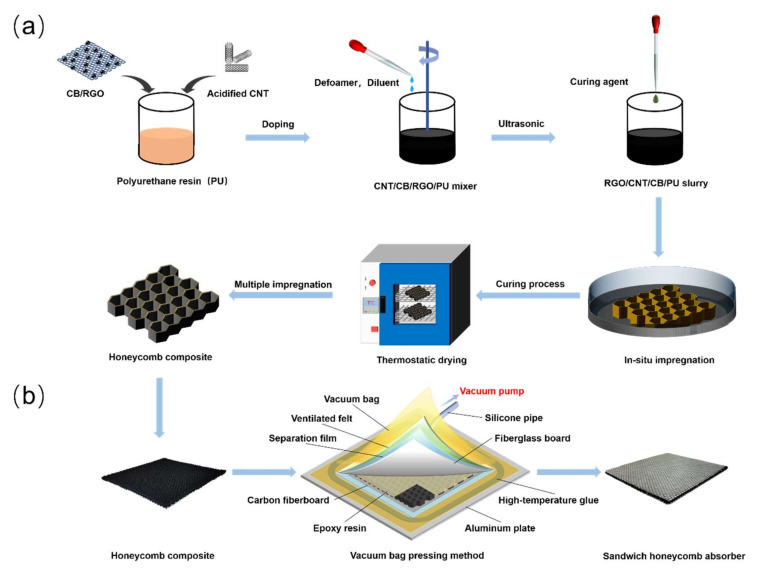
Illustration of (**a**) the HC composite fabrication progress and (**b**) the SHC absorber fabrication progress.

As shown in Figure 1a, the acidified CNTs and CB/RGO were fabricated through the method reported in our previous work [8,19]. CNTs and CB/RGO were mixed with PU using a high-speed mixer to form a uniform slurry. Each side of the HC core was placed into the slurry for 2 min and then pulled out vertically. Then, the HC core was dried rapidly at 120 °C to avoid the thickness gradient on the HC core caused by resin flow. Subsequently, the temperature was adjusted to 100 °C to solidify the impregnated layer on the HC core. A similar process can be repeated several times until the impregnation layer of the design thickness is obtained. The thickness of the impregnation layer is mainly controlled by the weight of the HC composite: the weight of the HC composite with an impregnation layer thickness of 33 μm is generally controlled at about 13 g. The HC composites were labeled as H_1_, H_2_, H_3_, and H_4_ according to the ratio of CNTs to CB/RGO in the slurry impregnated with the HC core as 1:1, 2:1, 3:1, and 4:1, respectively. multi-layer HC composites were numbered according to the order of bonding single-layer HC composites stacked from top to bottom. See Table 1 for details.

### 2.3. Preparation of CNT/CB/RGO/PU SHC Absorbers

As shown in Figure 1b, the SHC absorber is made of HC composite by the vacuum bagging method. Place ventilated felt, carbon fiberboard, HC composite, fiberglass board, separation film, ventilated felt, and vacuum bags on an aluminum plate in turn. Brush epoxy resin on HC composite, and connect vacuum pump after bonding vacuum bag to aluminum plate with high-temperature glue. Turn on the vacuum pump for pre-exhaust, checking for leaks (if leaks are found, use sealing tape to seal), and the vacuum degree is controlled at −0.09 MPa–−0.1 MPa. The SHC absorber was fabricated as shown in Figure 1b. Photos of HC core materials, HC composites, single-layer SHC absorbers, and multi-layer absorbers can be seen in Figure S1.

### 2.4. Analytical and Testing Instruments

The HC composite was characterized by scanning electron microscopy (SEM; Hitachi S-4800). The compressive test of the SHC absorber was carried out with a universal testing machine (WDW-10A). In addition, CEYEAR 3672C was used to measure the RL of the HC composites and SHC absorbers.

## 3. Results and Discussion

### 3.1. Morphological Analyses

**Figure 2:**
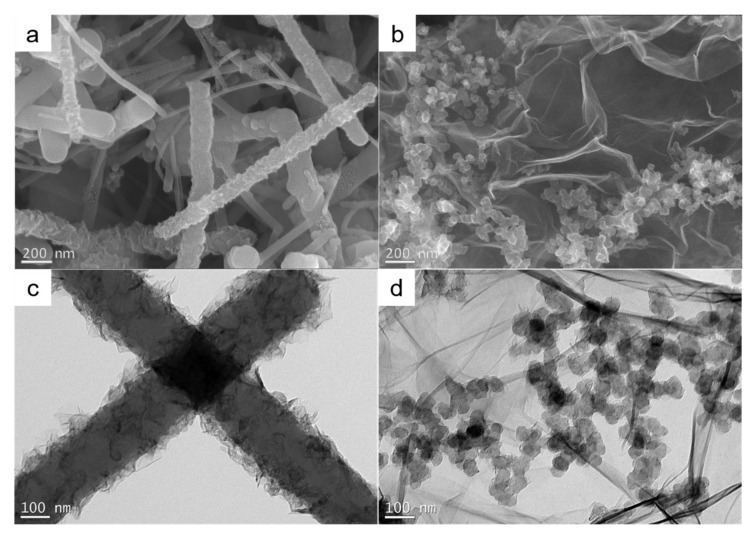
Microscopic morphology of CNT and CB/RGO (**a**) SEM image of CNT; (**b**) SEM image of CB/RGO; (**c**) TEM image of CNT; (**d**) TEM image of CB/RGO.

The EM wave-absorbing slurry impregnated by the HC composite was made of CNTs and CB/RGO homogeneously dispersed in PU. CNTs were further modified by acidification to improve their EM wave-absorption properties, and CB and RGO were made by one-pot method. Their microscopic morphology is shown in Figure 2. From Figure 2a,c, it can be seen that a large amount of flakes exist on the surface of the acidified modified CNTs. It can be speculated that this is due to the partial breakage of the C-C bond caused by the strong acid during the acidification treatment to form a large number of fine graphene flakes on the CNTs’ surface. In addition, the CNTs are MWCNTs, as evidenced by the graphene fragments on the surface and the tube diameter. CB/RGO prepared by one-pot method was formed by grafting CB onto RGO during the reduction of graphene oxide (GO) to RGO. From Figure 2b,d, it can be seen that CB is tightly attached to the surface of RGO in a grape shape. More information on the performance of this acid-modified CNT and CB/RGO can be found in the previous research results of our working group [8,19].

**Figure 3:**
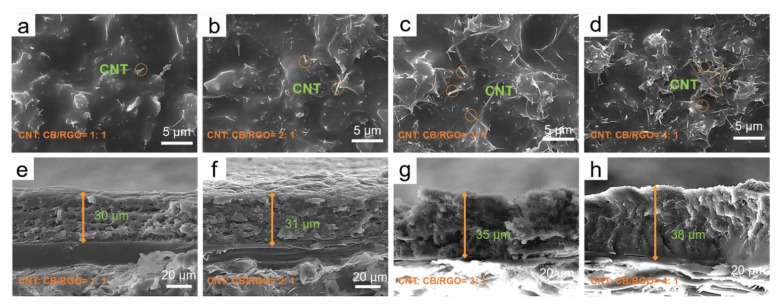
The micromorphology of the surface and cross-section of the impregnation layer of different slurries (**a**,**e**): CNTs:CB/RGO = 1:1; (**b**,**f**): CNTs:CB/RGO = 2:1; (**c**,**g**) CNTs:CB/RGO = 3:1; (**d**,**h**) CNTs:CB/RGO = 4:1.

It can be seen from Figure 3a–d that the surface of the impregnation layer gradually roughened due to the increased proportion of CNTs in CNT/CB/RGO/PU composites. On the surface, the micro-nanometer CB and the gentle RGO are not identifiable, but the rod-like CNTs are clearly visible and become increasingly dense as the proportion of CNTs in the composite increases. The most important thing is that CNTs are uniformly dispersed in the PU matrix, even when the mass ratio of CNTs to CB/RGO is 4:1 due to the acidification and ultrasonic separation of CNTs. This benefits CNT/CB/RGO/PU composites by having the advantages of multiscale carbon-based materials and creating microstructural conditions for the conduction loss and polarization loss of EM waves [20]. As shown in Figure 3e–h, the impregnation layer is attached to the HC core with a thickness range of 30 μm to 38 μm.

### 3.2. Dielectric Performance Analysis of CNT/CB/RGO/PU Composite

**Figure 4:**
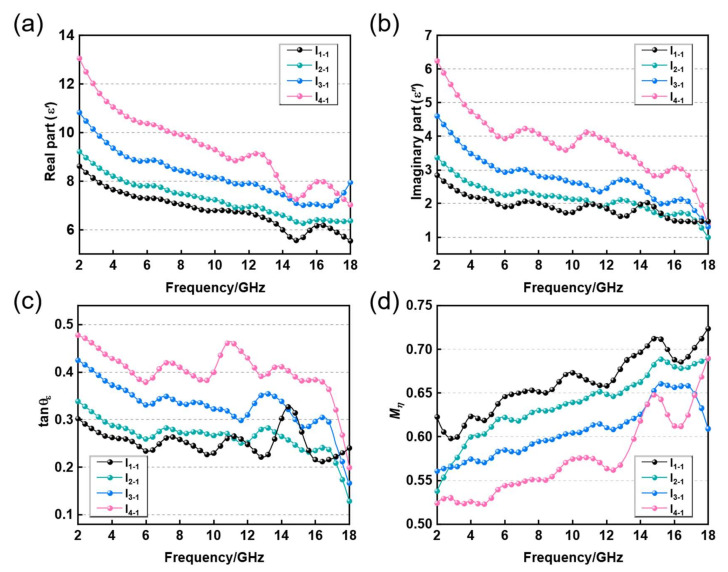
EM parameters of four kinds of impregnation layer material. (**a**) Real part of permittivity, (**b**) imaginary part of permittivity, (**c**) dielectric loss tangent angles, and (**d**) impedance matching coefficients ($M_{\eta }$).

In this paper, HC composites consist of an HC core and an impregnation layer of carbon-based absorbent. The HC core is made of EM loss-free aramid paper fibers with a hexagonal structure, which provides mechanical load bearing in the HC composite, while its unique structure facilitates multiple reflections of EM waves in the composite. As a multiscale carbon-based absorbing material, the CNT/CB/RGO/PU composite has good dielectric loss capability to provide EM absorption in HC composites. A vector network analyzer was used to measure the complex permittivity of the impregnation layer material in the 2–18 GHz frequency band. From Figure 4a,b, it can be seen that with the increased ratio of CNTs in the CNT/CB/RGO/PU composite, the real part ($\varepsilon^{\prime }$) and imaginary part ($\varepsilon^{\prime \prime }$) of the permittivity show a steady growth trend and decrease as the EM frequency rises. 

Based on the measured permittivity, the dielectric loss tangent angles ($\tan\sigma_{\varepsilon }$) and wave-impedance matching coefficients ($M_{\eta }$) of the four kinds of impregnation layer material were calculated and plotted. $\tan\sigma_{\varepsilon }$ indicates the dielectric loss capacity of the material: the larger the value, the better the dielectric loss performance [21], which can be calculated by Equation (1).

(1) $$\tan\sigma_{\varepsilon }=\frac{\varepsilon^{\prime \prime }}{\varepsilon^{\prime }}$$

$M_{\eta }$ is the degree of impedance matching. The closer the value is to 1, the higher the degree of impedance matching of the absorber [22], which can be calculated by Equation (2), where Re[X] is taken as the real part of complex X, $\varepsilon_{r}$ denotes the relative complex permittivity, and $\mu_{r}$ denotes the relative complex permeability. Since only the dielectric loss characteristics of carbon materials are considered in this paper, the default is $\mu^{\prime }=1$ and $\mu^{\prime \prime }=0$.

(2) Mη=2×Reμrεrμrεr2+1

As shown in Figure 4c,d, the H_1-1_ sample with the lowest CNT content has the worst dielectric loss capability and the strongest impedance matching, which is exactly the opposite for the H_4-1_ sample. In general, the CNT content is positively correlated with $\tan\sigma_{\varepsilon }$ and negatively correlated with $M_{\eta }$ of the impregnated layer [23,24]. This phenomenon may be caused by that the increased CNTs forming more conductive networks and improving the conduction loss capacity of the impregnation layer material. It could also be that the increased CNTs enrich the heterogeneous interfaces within the impregnated layer, thus enhancing the interfacial polarization [25,26,27].

**Figure 5:**
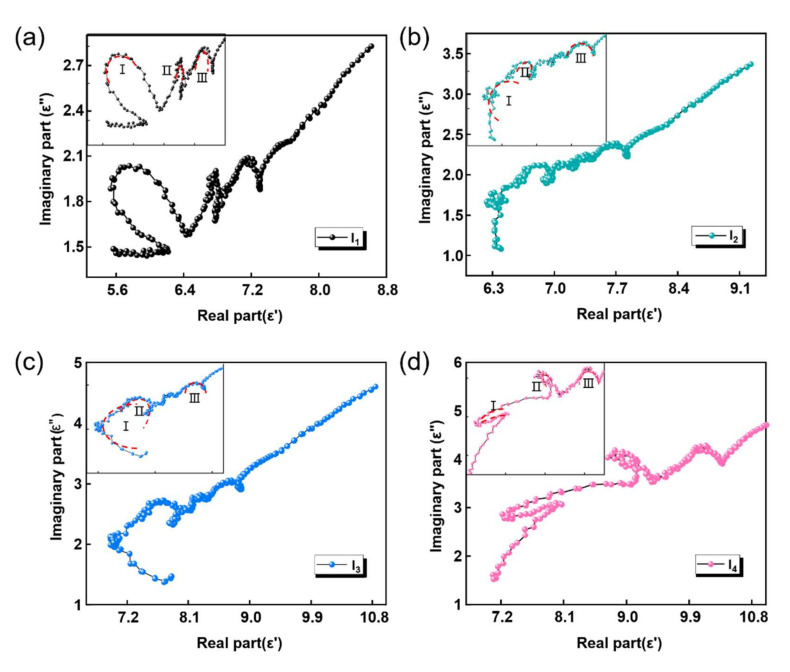
Plots of $\varepsilon^{\prime }$ versus $\varepsilon^{\prime \prime }$ for (**a**) I_1_, (**b**) I_2_, (**c**) I_3_, and (**d**) I_4_.

To further understand the polarization behavior of the impregnated layer material during EM absorption, the plot of $\varepsilon^{\prime }$ versus $\varepsilon^{\prime \prime }$ for all four samples is drawn in Figure 5a–d. Based on Debye relaxation, the relationship between $\varepsilon^{\prime }$ and $\varepsilon^{\prime \prime }$ can be described as Equation (3), where $\varepsilon_{s}$ and $\varepsilon_{\infty }$ represent the static dielectric constant and dielectric constant at infinite frequency, respectively. Therefore, the semicircle in the plot of $\varepsilon^{\prime }$ versus $\varepsilon^{\prime \prime }$ can signify the existence of the polarization process. As shown in Figure 5a–d, semicircles appear in all four images, proving the existence of polarization during the loss of EM waves in all four samples [28]. However, it can also be seen that the number of semicircles does not change as the proportion of CNTs increases, which means that the rise in CNTs does not significantly improve the polarization loss capacity of the impregnated layer material.

(3) $${\left(\varepsilon^{\prime }-\frac{\varepsilon_{s}+\varepsilon_{\infty }}{2}\right)}^{2}+{\left(\varepsilon^{\prime \prime }\right)}^{2}={\left(\frac{\varepsilon_{s}-\varepsilon_{\infty }}{2}\right)}^{2}$$

In summary, the increase in $\tan\sigma_{\varepsilon }$ and decrease in $M_{\eta }$ of the impregnated layer material are mainly due to the increased CNTs in the material, and enrich the conductive network, enhance conduction loss, and improve dielectric loss capability. However, at the same time, there is also an improvement in the conductivity of the material that makes the skinning effect of the material more obvious, resulting in lower impedance matching [29].

### 3.3. Absorption Properties of CNT/CB/RGO/PU HC Core Composite

#### 3.3.1. Analysis of Absorption Performance of Single-Layer HC Composite

The four single-layer HC composites were numbered H_1_, H_2_, H_3_, and H_4_ according to the proportion of CNTs in the HC composite impregnating slurry. The EM reflection-loss testing and analysis results of the four HC composite materials are as follows.

**Figure 6:**
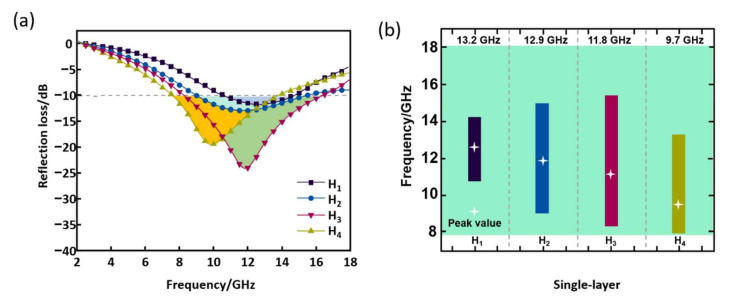
RL of single-layer HC composite. (**a**) RL curves for different ratios, (**b**) frequency range and bandwidth of EM wave absorption for each sample.

According to microwave-absorption theory, the performance of absorbing materials is mainly determined by the degree of impedance matching and EM wave-attenuation ability [30]. The ideal absorbing material must have a good synergy between these two factors. As shown in Figure 6, the intensity of the RL curve of the single-layer HC composite increases and then decreases as the proportion of CNTs within the impregnation layer increases. The best EM absorption performance is H_3_: RL_min_ is −25.2 dB with an effective absorption bandwidth of 7.34 GHz (8.3–15.6 GHz). This phenomenon suggests that the formation of conductive paths favors the increase of EM absorption in the composites as the mass ratio of CNTs increases [31,32,33,34]. However, it also decreases the impedance matching degree, making it difficult for EM waves to enter the material’s interior, thus preventing the effective absorption of EM waves [35,36,37].

#### 3.3.2. Analysis of Loss Mechanism of Single-Layer HC Composite

**Figure 7:**
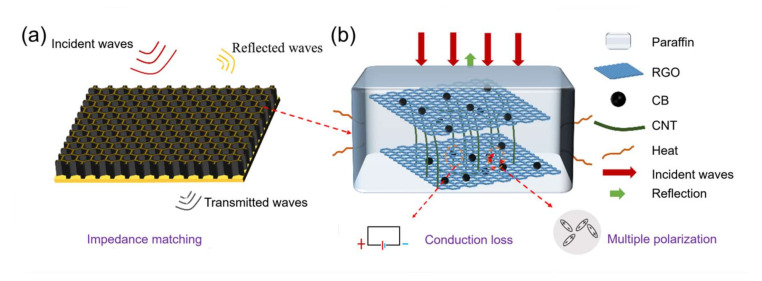
The absorption mechanism of the HC composite: (**a**) macroscopic field; (**b**) microscopic field.

The schematic diagram of the absorption mechanism of the single-layer HC structure is shown in Figure 7. From Figure 7a, it can be seen that impedance matching played an essential role in the HC composite. The porous structure of the HC composite allows more EM waves to enter the interior of the composite, increasing the incidence rate. The hexagonal structure of porous HC core makes the EM wave scatter multiple times inside the HC composite, which effectively increases the probability of the interaction between the EM wave and the lossy particles. In addition, as shown in Figure 7b, the ternary carbon materials inside the impregnation layer are superimposed on each other to form a large number of conductive networks and heterogeneous interfaces, resulting in excellent conduction loss and interface polarization. At the same time, a large number of groups and defects exist on the surface of acidified CNTs and CB/RGO, which can easily become the center of dipole polarization [8,38]. The multiscale carbon-based absorbent used in this paper further increases the absorption channels, which is beneficial to the absorption performance of the HC composite. Therefore, for the HC structure material, the wave-absorbing performance results from the promotion of many factors. The critical point is how to coordinate the structural parameters of the HC core and the EM properties of the impregnation layer material. 

#### 3.3.3. Analysis of Absorbing Performance of Multi-Layer HC Composite

The prepared single-layer HC composite has good absorbing properties, but its effective absorbing bandwidth is still not ideal. In order to further improve the wave-absorbing properties of HC composites, structural design is explained out in this section. Currently, the structural design of multifunctional materials mainly includes two methods: graded impedance design and graded medium design. Gradient impedance absorber, also known as Jaumann absorber [39], consists of several resistive films placed on different layers and a lossless substrate filled between every two resistive films [40,41,42]. Graded medium absorbers, also known as gradient absorbers, are made of layers of lossy materials with gradient designs to improve wave-impedance matching. The incident wave attenuates as it propagates through these lossy layers [43,44]. It was obvious for HC composites that this approach of graded medium design was most suitable.

According to the impedance matching degree and dielectric loss tangent of the four proportions of impregnation layer materials, the design of the multi-layer HC composite material in this paper is shown in Table 1.

**Figure 8:**
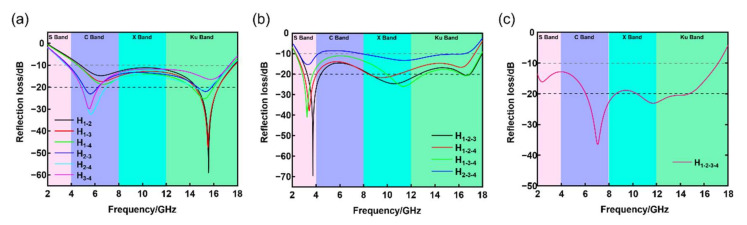
Reflection loss of multi-layer HC composites. (**a**) Double-layer, (**b**) triple-layer, (**c**) quadruple-layer.

As shown in Figure 8a, the RL curves of the double-layer HC composite shift their harmonic peaks to the left when the permittivity of the impregnation material of the bottom layer increases. This phenomenon is consistent with the description of dielectric loss materials in the quarter-wave cancellation theory [45]. The double-layer HC composites with the best absorbing performance were samples H_2-4_: the effective absorption bandwidth is about 14.1 GHz (3.6–17.7 GHz) and the maximum absorption intensity −34 dB. Figure 8b shows the RL of the triple-layer HC composite. The RL value of sample H_1-3-4_ in the 2.3–18 GHz range is less than −10 dB, which is better than the absorbing performance of other triple-layer HC composites. Figure 8c shows a quadruple-layer gradient HC composite’s absorption bandwidth is 14.2 GHz, and the maximum absorbing intensity is −37.2 dB. Compared with the triple-layer HC composite, the absorbing bandwidth and intensity are not significantly enhanced. Instead, the absorbing bandwidth becomes narrower, showing that the excessive dielectric properties harm absorbing performance. In general, among the prepared multi-layer HC composites, the triple-layer HC composites H_1-3-4_ with an absorption bandwidth of 15.8 GHz and an absorption peak of −37.2 dB are the samples with the best absorption performance.

#### 3.3.4. Analysis of Loss Mechanism of Multi-Layer HC Composite

**Figure 9:**
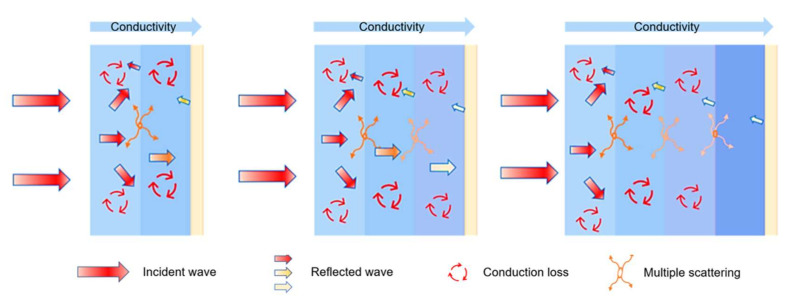
The wave-absorption mechanism of the multi-layer HC composites.

On the basis of EM wave loss of single-layer HC composite material, the designed multi-layer HC composite material has high impedance matching and strong EM wave loss ability. This advantage mainly comes from the graded medium design of the multi-layer HC composites. As shown in Figure 9, the incident wave first makes contact with the low CNT ratio HC single layer with good impedance matching before entering the multi-layer HC composite, which allows more EM waves to enter the interior. The impregnation layer material is responsible for attenuating EM waves in the multi-layer HC composite. At the same time, multiple scattering occurs between the layers to increase the EM wave propagation path. As the number of layers increases, there are more opportunities for EM waves to interact with the composite material, which means that the EM waves can be repeatedly attenuated and converted into heat dissipation. Two prerequisites for an ideal absorber are strong EM wave loss capability and high impedance matching, both of which are met here. Therefore, the multi-layer HC composite attained good absorbing properties [46,47,48].

### 3.4. Analysis of Absorption Performance of Sandwich Honeycomb Absorber

We prepared single-layer and multi-layer HC composites with the best absorbing properties into SHC absorbers. By the vacuum bagging method, fiberglass board and carbon fiberboard are respectively bonded on the upper and bottom layer surfaces of the HC composite to form the SHC absorber. A physical sample image of the multi-layer SHC can be seen in Figure S1. 

**Figure 10:**
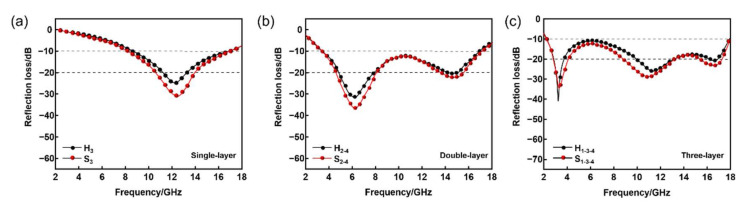
Comparison of RL between HC composites and SHC absorber. (**a**) Single-layer, (**b**) double-layer, (**c**) triple-layer.

As shown in Figure 10, the absorbing properties of the single-layer and multi-layer SHC absorbers are improved compared with their corresponding HC composites. In Figure 10a, the RL intensity of the single-layer SHC absorber is increased from −25.2 dB to −33 dB, and the effective absorption bandwidth is broadened from 7.3 GHz to 7.5 GHz. Similarly, it can be seen from Figure 10b,c that the absorption intensity of the double-layer and triple-layer SHC absorbers is enhanced compared with their corresponding HC composites. The maximum intensity of the triple-layer SHC absorber RL is −35 dB, and RL < −10 dB in the frequency range of 2.2 to 18 GHz. It can be speculated that the reason for this enhancement is that the fiberglass board and the carbon fiberboard are totally transmissive and perfectly reflective for EM waves, respectively. 

### 3.5. Mechanical Properties of SHC Absorber

The introduction of fiberglass board and carbon fiberboard will inevitably improve the mechanical properties of HC composites. Therefore, improvement of the mechanical properties of the SHC absorber by the CNT/CB/RGO/PU impregnation layer is mainly studied with the compression test of pristine SHC (without impregnation layer) and impregnated SHC. The flexibility performance of HC and the compression testing process can be seen in Figure S2.

**Figure 11:**
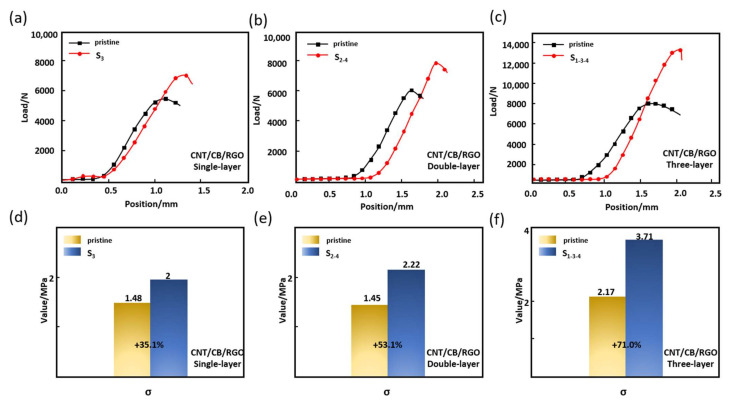
Mechanical properties of sandwich HC absorber. (**a**–**c**) The load-deformation curves. (**d**–**f)** the compression stress.

Figure 11 shows the mechanical properties of the SHC absorber. Figure 11a–c are the load-deformation curves of the SHC absorber, and Figure 11d–f show the compression stress of SHC absorber. It can be seen from the SHC absorber that the fracture deformation and compression stress are enhanced. The three SHC absorbers’ compressive stress is increased by 35.1%, 53.1% and 71.1%, respectively. The above results show that the SHC absorber coated with the CNT/CB/RGO/PU composite can withstand greater compression force and compressive stress than the pristine SHC absorber. Therefore, it can be concluded that the SHC absorber effectively integrates mechanical loading and EM absorption, and is a promising multifunctional material.

## 4. Conclusions

The HC composites impregnated with CNT/CB/RGO/PU slurry were successfully prepared, and the thickness of the impregnation layer was 33.5 μm on average. The analysis of the dielectric properties of the impregnation layer material found that as the proportion of CNTs in it increased, the dielectric loss properties of the material improved and the impedance matching decreased, which was due to the enhanced conduction loss. The multi-layer HC composites designed by the gradient medium principle have good wave-absorbing properties. The single-layer, double-layer, and triple-layer HC composites with the best wave-absorbing properties were prepared for SHC absorbers by the vacuum bagging method. By analyzing the absorbing performance of the SHC absorber, it is found that the best maximum RL intensity of the triple-layer absorbing performance is −35 dB, and RL < −10 dB in the frequency range of 2.2–18 GHz. Furthermore, the average compressive stress of the SHC absorber reached 3.71 MPa, which is similar to the compressive stress of aluminum HC panels for aviation. The radar-absorbing HC composite is a promising candidate for military stealth applications from a practical perspective.

## Figures and Tables

**Table 1:** Stack details and thicknesses of multi-layer CNT/CB/RGO/PU HC composite.

Sample Code	Thickness	Stack Number (from Top to Bottom)
#1-2	10 mm	1, 2
#1-3	10 mm	1, 3
#1-4	10 mm	1, 4
#2-3	10 mm	2, 3
#2-4	10 mm	2, 4
#3-4	10 mm	3, 4
#1-2-3	15 mm	1, 2, 3
#1-2-4	15 mm	1, 2, 4
#1-3-4	15 mm	1, 3, 4
#2-3-4	15 mm	2, 3, 4
#1-2-3-4	20 mm	1, 2, 3, 4

## Data Availability

Not applicable.

## Supplementary Materials

The following supporting information can be downloaded at: https://www.mdpi.com/article/10.3390/nano12152622/s1, Figure S1: (**a**) Original HC core material; (**b**) HC composite; (**c**) SHC absorber; (**d**) multi-layer SHC absorber; Figure S2: (**a**) Normal state of HC composite; (**b**) Bending state of HC composite; (**c**) Compression test of HC composite.
