# Supplementary material for: Lightweight and Compression-Resistant Carbon-Based Sandwich Honeycomb Absorber with Excellent Electromagnetic Wave Absorption
Source: Nanomaterials (Basel). 2022 Jul 29;12(15):2622. doi: 10.3390/nano12152622 (PMC9370204; doi:10.3390/nano12152622)
Supplement: Supplementary file 1 [file nanomaterials-12-02622-s001.zip › nanomaterials-1837081-supplementary.pdf]

nanomaterials

Supplementary Materials

Lightweight and Compression-Resistant Carbon-Based Sandwich Honeycomb Absorber with Excellent Electromagnetic Wave Absorption

Song Bi [1,*], Yongzhi Song [1], Genliang Hou [1], Hao Li [1], Nengjun Yang [1] and Zhaohui Liu [2,*]

[1] 304 Department, Xi'an Research Institute of High-Tech, Xi'an 710025, China; syz237530783@163.com (Y.S.); hougenliang@163.com (G.H.); 376467729lihao@163.com (H.L.); yangbcpl@163.com (N.Y.)
[2] College of Weapon Science and Technology, Xi'an Technological University, Xi'an 710025, China
* Correspondence: xiaozhu-youyou@163.com (S.B.); lzh5011@163.com (Z.L.)

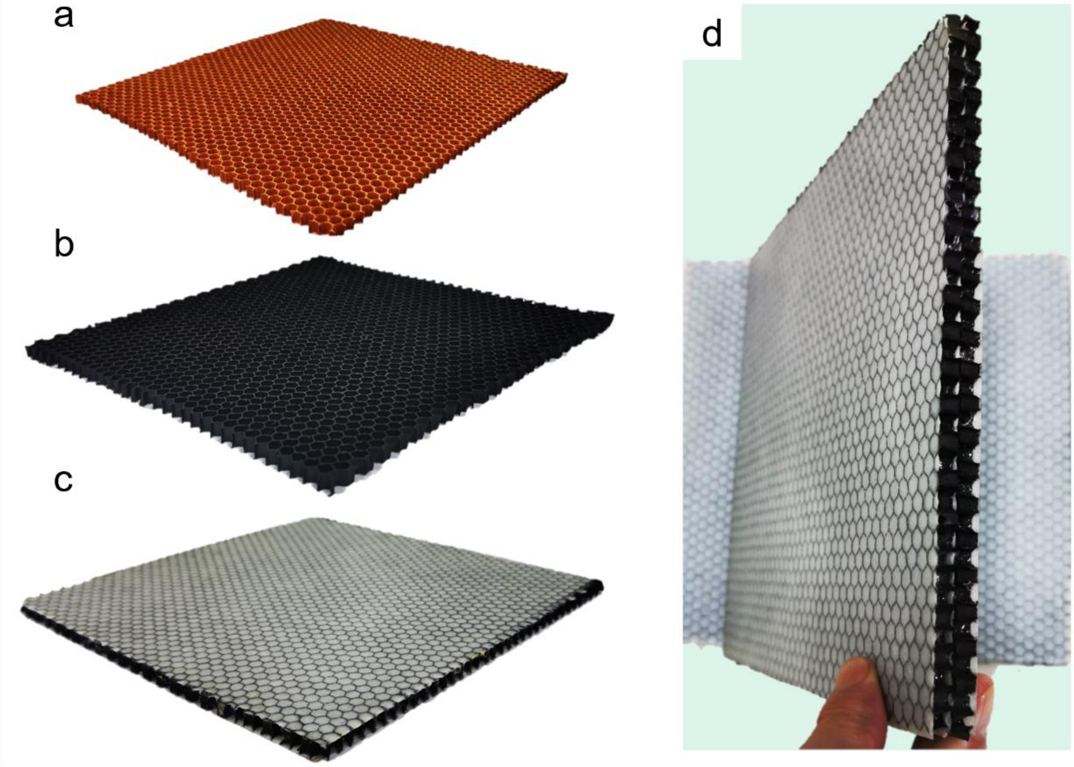

Figure S1. (**a**) Original HC core material; (**b**) HC composite; (**c**) SHC absorber; (**d**) multi-layer SHC absorber.

The original HC core was impregnated with CNTs/CB/RGO/PU EM wave absorbing slurry to form the HC composite as shown in Figure S1a-d. From Figure S1b, it can be seen that the slurry only adheres to the HC core wall to form the impregnation layer material, retaining the original unique structural characteristics of HC. Figure S1c is a single-layer SHC absorber made by bonding fiberglass board and carbon fiberboard on the top and bottom surfaces of HC composite material by vacuum bagging method. Figure S1d shows a double-layer of SHC absorber, prepared by vacuum bagging method according to the stacking method in Table I. Similarly, the multi-layer SHC absorber are vacuum bagged according to the corresponding stacking method in Table 1.

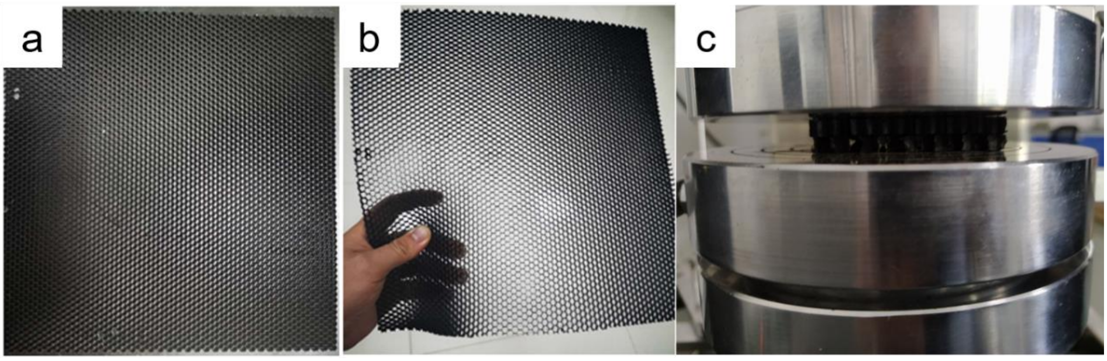

Figure S2. (**a**) Normal state of HC composite; (**b**) Bending state of HC composite; (**c**) Compression test of HC composite.

As shown in Figure S2, the HC composite in its normal state is naturally stretched. The lightweight and flexible HC composite has a great degree of bendability and returns to normal immediately after relaxation. However, due to its hexagonal structure, the HC composite has a very high compressive strength.Therefore, HC composites are well suited as structural wave absorbing materials in stealth vehicles, assuming the functions of structural load-bearing and wave absorber.
